# Transcriptome research identifies four hub genes related to primary myelofibrosis: a holistic research by weighted gene co-expression network analysis

**DOI:** 10.18632/aging.203619

**Published:** 2021-10-11

**Authors:** Weihang Li, Yingjing Zhao, Dong Wang, Ziyi Ding, Chengfei Li, Bo Wang, Xiong Xue, Jun Ma, Yajun Deng, Quancheng Liu, Guohua Zhang, Ying Zhang, Kai Wang, Bin Yuan

**Affiliations:** 1 Department of Orthopaedics, Xijing Hospital, The Fourth Military Medical University, Xi’an 710032, People’s Republic of China; 2 Department of Intensive Care Unit, Nanjing First Hospital, Nanjing Medical University, Nanjing 210006, Jiangsu Province, China; 3 Department of Aerospace Medical Training, School of Aerospace Medicine, Fourth Military Medical University, Xi’an 710032, Shaanxi, China; 4 Department of Hematology, Daxing Hospital, Xi’an 710016, Shaanxi, China; 5 Department of Spine Surgery, Daxing Hospital, Xi’an 710016, Shaanxi, China

**Keywords:** bioinformatics, WGCNA, myeloproliferative, neoplasm, myelofibrosis

## Abstract

Objectives: This study aimed to identify specific diagnostic as well as predictive targets of primary myelofibrosis (PMF).

Methods: The gene expression profiles of GSE26049 were obtained from Gene Expression Omnibus (GEO) dataset, WGCNA was constructed to identify the most related module of PMF. Subsequently, Gene Ontology (GO), Kyoto Encyclopedia Genes and Genomes (KEGG), Gene Set Enrichment Analysis (GSEA) and Protein-Protein interaction (PPI) network were conducted to fully understand the detailed information of the interested green module. Machine learning, Principal component analysis (PCA), and expression pattern analysis including immunohistochemistry and immunofluorescence of genes and proteins were performed to validate the reliability of these hub genes.

Results: Green module was strongly correlated with PMF disease after WGCNA analysis. 20 genes in green module were identified as hub genes responsible for the progression of PMF. GO, KEGG revealed that these hub genes were primarily enriched in erythrocyte differentiation, transcription factor binding, hemoglobin complex, transcription factor complex and cell cycle, etc. Among them, EPB42, CALR, SLC4A1 and MPL had the most correlations with PMF. Machine learning, Principal component analysis (PCA), and expression pattern analysis proved the results in this study.

Conclusions: EPB42, CALR, SLC4A1 and MPL were significantly highly expressed in PMF samples. These four genes may be considered as candidate prognostic biomarkers and potential therapeutic targets for early stage of PMF. The effects are worth expected whether in the diagnosis at early stage or as therapeutic target.

## INTRODUCTION

World Health Organization (WHO) revised and updated the hematopoietic tumor classification system in 2016, which confirmed seven major categories of myeloid malignancies including acute myeloid leukemia (AML) and related neoplasms, myeloproliferative neoplasms (MPN), myelodysplastic syndromes (MDS), mastocytosis, eosinophilia-associated myeloid/lymphoid neoplasms with specific mutations, and MDS/MPN overlap and myeloid neoplasms with germline predisposition [1], among which MPNs are clonal hematopoietic disorders characterized by excessive production of differentiated hematopoietic cells in chronic phase [2]. The Philadelphia-negative MPNs contained 3 major diseases including polycythemia vera (PV), essential thrombocythemia (ET) and primary myelofibrosis (PMF). Among MPNs, PMF is the most essential neoplasms which could evolve from other MPNs such as PV and ET. Later in their courses, both PV and ET disorders may evolve into myelofibrotic phases termed “post polycythemia vera myelofibrosis” or “post-essential thrombocythemia myelofibrosis”, respectively [3, 4]. In this situation, PMF is referred to as post-PV MF or post-ET MF.

PMF is a nascent, myeloproliferative, neoplastic disorder characterized by clonal proliferation of myeloid cells in bone marrow, which could result in fibrosis and lead to the devastation of healthy marrow [3, 5, 6]. Common clinical manifestations of PMF include marked splenomegaly, anemia as well as constitutional symptoms such as fatigue, fever, weight loss and night sweats. Patients with severe symptoms of PMF may show upper abdomen flatulence feeling, bone pain, bleeding, and cachexia, etc. [7–9]. Besides, the overall treatment and prognosis of PMF is generally poor. Epidemiology findings showed that the estimated prevalence of PMF is between 4 - 6/100000 people per year, with a median survival time of 15 years for patients younger than 60 and 6 years for patients older than 60, respectively [10, 11]. Chemotherapy is regarded as a treatment of PMF, however, chemotherapy, including JAK2 inhibitors does not provide a promising prospective view at this moment [12, 13], which may result from inadequate exploration of genes in the occurrence and development of PMF. Consequently, there is an urgent need to discover novel targeted chemotherapy from exploring therapeutic and diagnostic targets of PMF.

Recent decades, bioinformatics combined with microarray technology have displayed a promising view for analyzing molecular and genetic mechanisms of malignant neoplasms [14–16], which prompted study on the initiation, development, and metastasis of tumor. Weighted Gene Co-expression Network Analysis (WGCNA) is a holistic and systematic biological method, which is used to construct network analysis for exploring the correlation between genes and given features [17, 18]. The mathematical principle of WGCNA was firstly proposed in 2005 [19], and the algorithm was implemented in R environment in 2008, namely “WGCNA package” [18]. Recent years, WGCNA has been witnessed for extensive application in many fields, such as identifying the candidate biomarkers from uveal melanoma, breast cancer and adrenocortical carcinoma, etc. [20–24]. With the assistance of WGCNA, researchers could explore potential mechanisms among highly co-related genes and discover novel diagnostic or therapeutic targets from gene cluster associated with disease. To the best knowledge, the application of WGCNA in the identification of PMF has rarely been reported, so this study may provide a new thinking to analyze PMF.

In the current study, microarray dataset GSE26049 has been analyzed to construct a weighted co-expression network and to find a module with specific interest to the development of PMF, then the most related module was applied by functional enrichment analysis including GO, KEGG, and GSEA, to discover molecular function and aberrant regulated pathways. Next, PPI network analysis was conducted on the interested module, to visualize the relationships between co-related genes and identify the top related genes. A series of validation methods including machine learning, principal component analysis, qRT-PCR, etc., were performed to verify the conclusions. Results may contribute to assess the prognosis of PMF and ultimately offer new ideas in the treatment of PMF.

## RESULTS

### Profile’s quality control and preprocess

**Figure 1:**
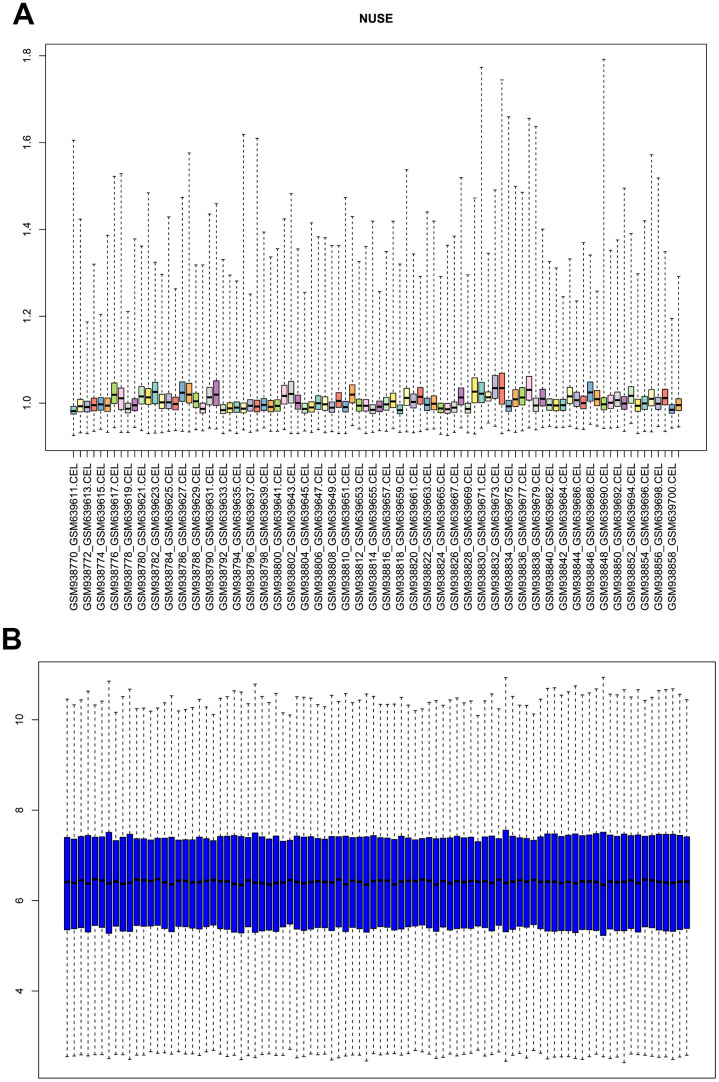
(**A**) Normalized unscaled standard error (NUSE) plot of GSE26049 for quality control. (**B**) box plot of gene expression level in GSE26049 after RMA background correction and normalization.

The microarray dataset GSE26049 was applied in this study, gene expression profiles of normal samples and different tumor samples (total n=90) were normalized and generated. Unqualified samples were eliminated according to the quality control of these gene chips, and standard samples were filtered by calculating the normalized unscaled standard errors (NUSE), NUSE plot was displayed for quality check (Figure 1A). Results illustrated that all these 90 samples had a good chip quality and could be used for further research. Then RMA algorithm was performed for data preprocessing, the boxplot of normalized and background corrected value was plotted (Figure 1B), results showed that the median amount of gene expression in each sample was on a straight line, indicating that the preprocessed data was served as standard data and could be analyzed in the following study.

### Construction of weighted gene co-expression network

**Figure 2:**
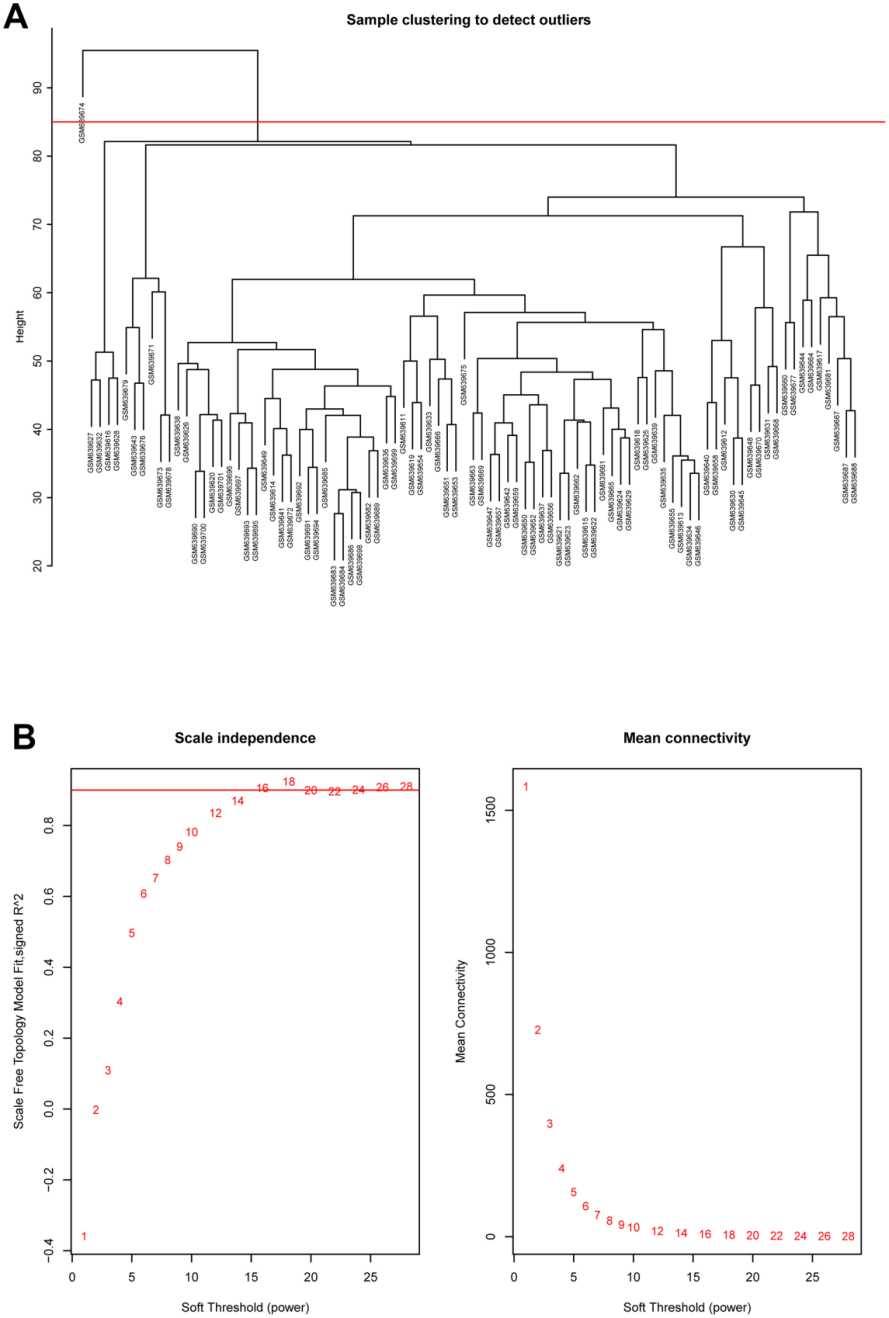
(**A**) Cluster dendrogram of samples in GSE26049 to detect outliers. The dendrogram branches represented the clustered samples. (**B**) Selection of soft threshold power value through WGCNA analysis. The left panel showed the scale-free model fit index (y axis) as function of soft thresholding power value (x axis). Red line represented the y-axis value. Right panel displayed the mean connectivity (y axis) as a function of soft thresholding powers (x axis).

Hierarchical clustering analysis was applied to check the heterogeneity of each sample to detect and remove the outliers (Figure 2A). Red line, namely cutoff threshold height, was set as 85 to filter outliers. Finally, sample GSM639674 was excluded, and the gene expression matrix containing 20482 genes in the rest of 89 samples were used for WGCNA analysis. Then the optimal soft threshold power was calculated, as presented in Figure 2B, when the soft threshold power β reached 16, the scale free topology model fit index (R^2) was higher than 0.90 and mean connectivity was infinitely approaching 0. As a result, power 16 was selected as the optimal soft threshold power value.

**Figure 3:**
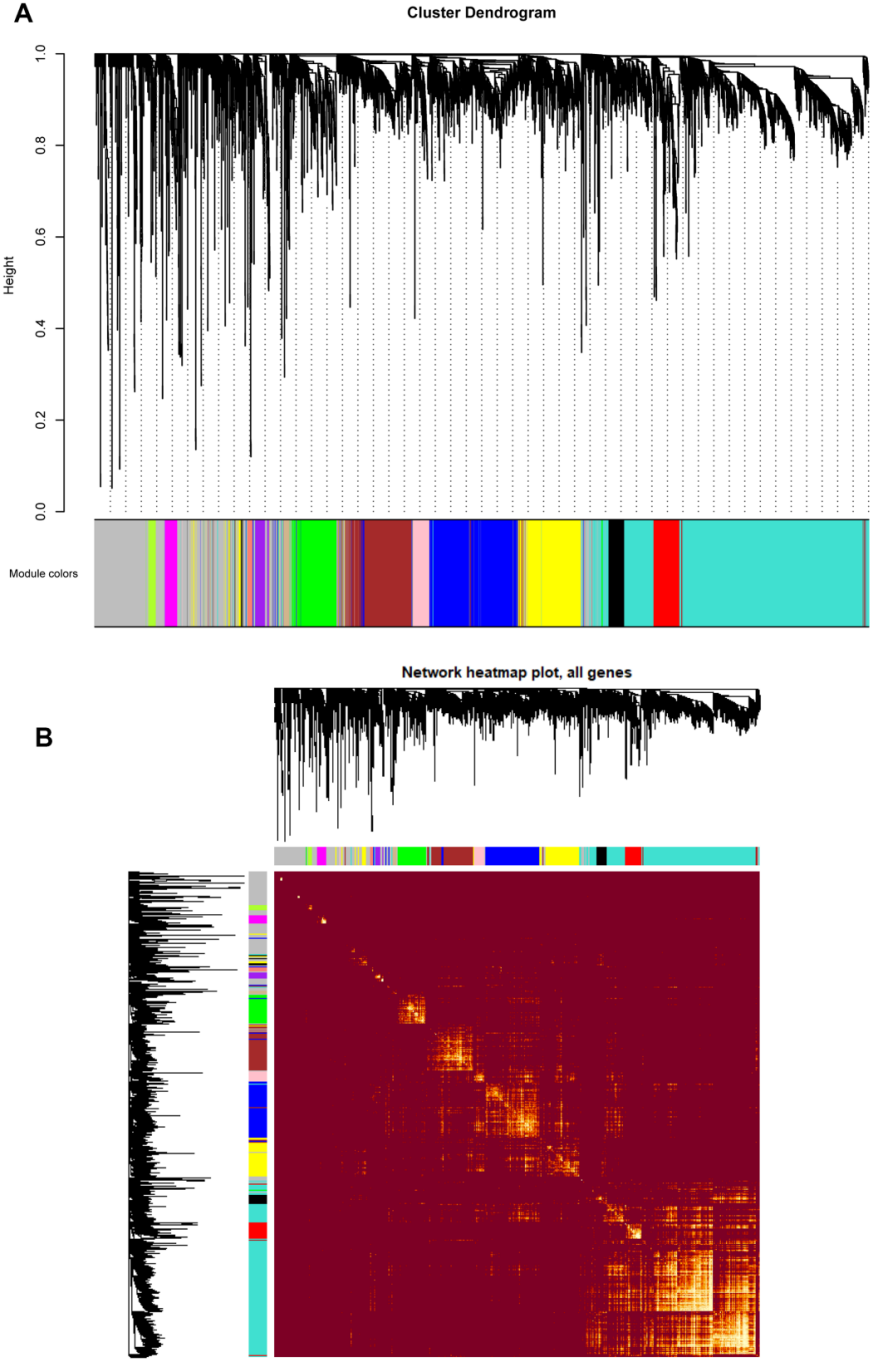
(**A**) Cluster dendrogram of all genes enriched based on the dissimilarity measure and the assignment modules. Highly interconnected groups of genes were clustered. Every color below represented one co-expression module, totally 14 distinct modules were identified with the hierarchical clustering tree analysis. (**B**) Topological overlap heatmap of the gene co-expression network. Each row and column represented a gene. Light color indicated high topological overlap and dark color represented low topological overlap. Different colors on x and y axis indicated different modules. The dendrogram suggested that the clustering of these genes was based on the similarity of their gene expression levels.

Based on the co-expression relationships, hierarchical clustering analysis was then performed to obtain the weighted co-expression network (Figure 3A), results illustrated that 14 distinct co-expression modules, characterized by their unique module color, were identified and clustered. The module colors included salmon, tan, blue, purple, yellow, brown, pink, magenta, black, red, turquoise, green, green-yellow, and grey, respectively. Interaction relationships of these 14 co-expression modules in all genes were shown in Figure 3B, topological overlap heatmap revealed that each co-expression module could independently validate each other in the network, and dendrogram branches indicated that genes in each module were highly heterogenous. Consequently, it was necessary to further identify the interested modules in each subgroup.

### Key module identification of PMF

**Figure 4:**
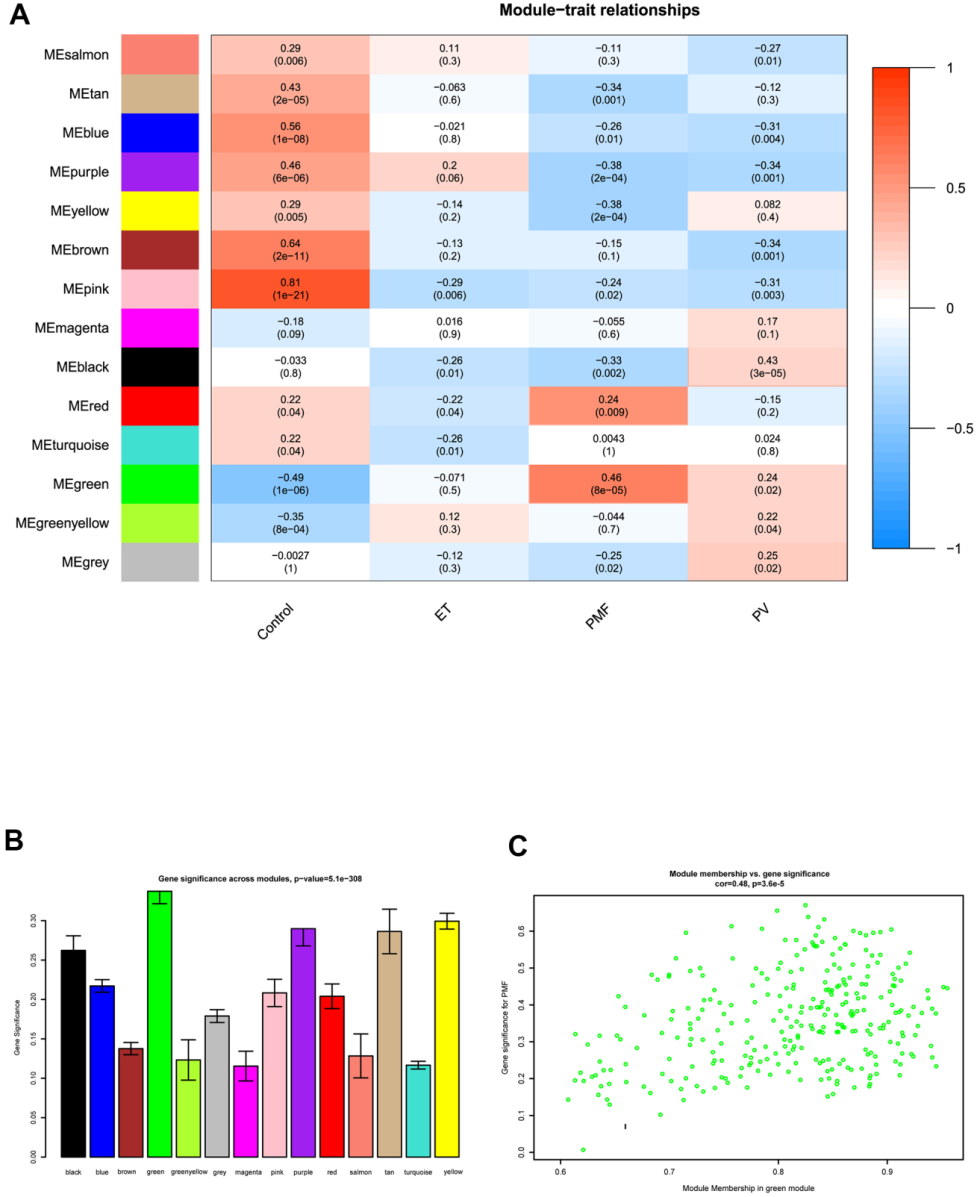
(**A**) The module-trait relationship heatmap between MEs and features. Each row corresponded to a module eigengene and column to a feature. The first column was normal samples; the second column was essential thrombocythemia; the third column was primary myelofibrosis; and the fourth column was polycythemia vera. Each square contained corresponding correlation (first line) and p value (second line). The left side of heat map indicated the module name, right side of heat map indicated the colors of correlation (blue represented negatively correlated, red represented positively correlated). (**B**) Histogram of gene significance across modules in PMF group. (**C**) The correlation between gene significance and module membership in green module. The y-axis indicated gene significance in green module, and x-axis represented the module membership in green module.

After obtaining WGCNA network data, the module-trait relationships heatmap was then performed. The interaction relationships between each module and each different feature including control, PV, ET, and PMF subgroups were fully assessed (Figure 4A, 4B). X axis indicated the features and Y axis represented the module, from the heatmap and histogram, results demonstrated that black module was significantly associated with PV (P = 3e-05), no co-expression module was significantly related to ET (P > 0.05). As for PMF, results visualized that correlations of PMF samples in the green (P = 8e-05) and red (P = 0.009) modules had strong positive correlation compared to normal samples, while purple (P = 2e-04) and yellow (P = 2e-04) modules had a strong negative correlation, indicating that modules green, red, purple and yellow could significantly differentiate the PMF samples from the normal samples, and genes in these modules could promote or suppress in the progression of PMF. Among them, green module had the strongest correlation (r = 0.46) and the lowest P value (p = 8e-5), elucidating green module was most correlated with PMF, genes in green module were essential in the pathogenesis and oncogenesis of PMF. Besides, Module Membership (MM) versus Gene Significance (GS) scatter plot (MM-GS plot) illustrated that MM was highly correlated with GS in green module (cor= 0.48, p = 3.6e-5) (Figure 4C).

**Figure 5:**
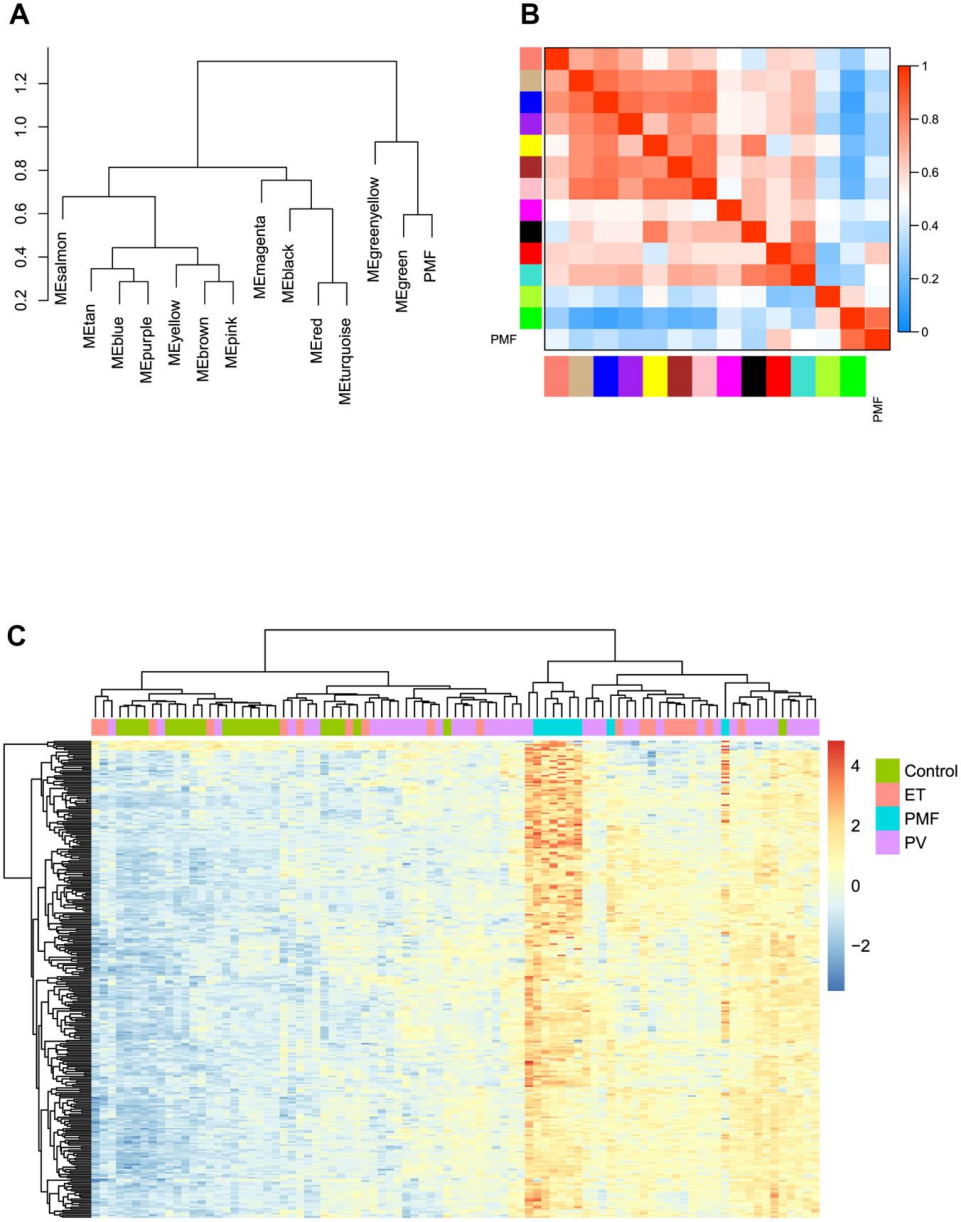
(**A**) The cluster dendrogram of adjacencies in the eigengene network. (**B**) The heat map of adjacencies in the eigengene network. Blue represented a negative correlation, while red represented a positive correlation. (**C**) Green module genes expression heat map of different groups (Control, ET, PV and PMF).

Module eigengene adjacency was subsequently calculated to cluster and assess the identified modules with PMF feature, heatmap was plotted to depict their interactions between modules and PMF subgroup. Based on the dendrogram, result illustrated that the PMF subgroup clustered with green module, implying that these two eigenvalues had a highly correlations with each other (Figure 5A). As for heat map, each module exhibited their independent validation to the other. The color depth (red to blue) represented the different strength of co-expression interactions, results verified that PMF subgroup had a highly correlations with green module (Figure 5B).

Then we performed hierarchical clustering analysis based on gene expression value in green module between four subgroups: control, PV, ET and PMF. As presented in Figure 5C, results demonstrated that the genes in green module could significantly differentiate PMF group from other three groups, and PMF samples clustered tightly with each other compared to other groups, which was consistent with above results. Consequently, green module was identified as key module accounting for the progression of PMF and subsequent research was pooled based on the green module genes.

### Functional and pathway enrichment analysis

**Figure 6:**
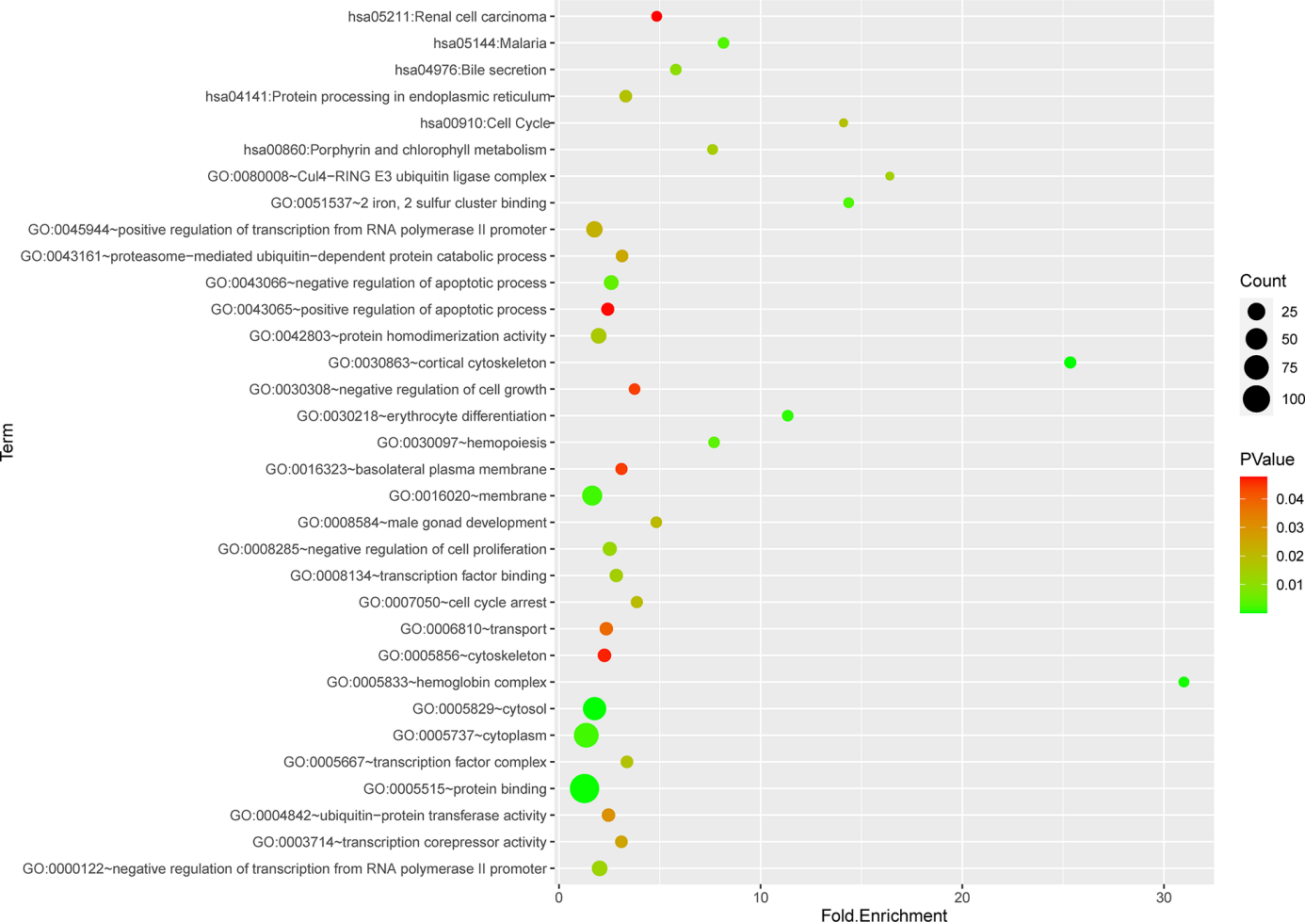
Functional and pathway enrichment analysis of genes in green module.

**Figure 7:**
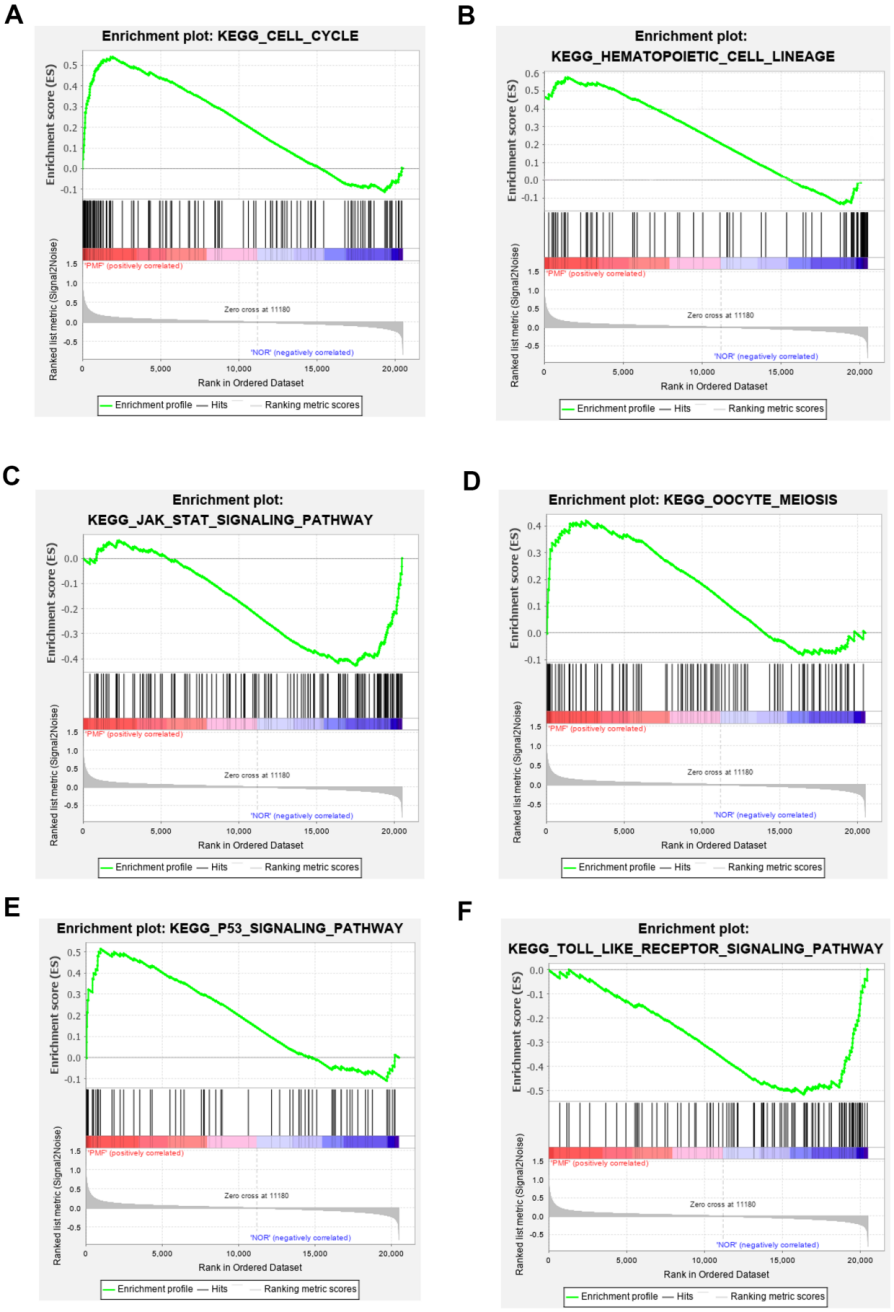
(**A**–**F**) Gene set enrichment analysis of KEGG pathways in green module.

Totally green module contained 217 genes (as shown in Supplementary Table 1), to obtain a comprehensive understanding of the biological functions and aberrant signaling pathway of the genes in green module, GO, KEGG and GSEA methods were conducted. These genes were uploaded into DAVID database for BPs, MFs, and CCs analysis, the detailed results of GO, KEGG was shown in Table 1 and Figure 6. GO results indicated that these genes were mainly associated with several BPs including erythrocyte differentiation, hemopoiesis, positive regulation of transcription from RNA polymerase II promoter and negative regulation of apoptosis process, etc.; CCs including cytosol, hemoglobin complex, transcription factor complex, etc.; and several MFs such as protein binding, transcription factor binding, heme binding, etc. KEGG analysis revealed that some signaling pathways were significantly changed, such as cell cycle, protein processing in endoplasmic reticulum, bile secretion and porphyrin metabolism. As shown in Figure 7A–7F, GSEA results indicated that the mutual genes in green module were commonly evolved in critical signaling pathways that were correlated with carcinogenesis of tumor, including cell cycle, hematopoietic cell lineage, JAK-STAT signaling pathway, oocyte meiosis, P53 signaling pathway and toll-like receptor signaling pathway, etc.

**Table 1:** Functional and pathway (GO, KEGG) enrichment analysis of the genes in green module.

**Category**	**Term**	**Count**	**%**	**P Value**
GOTERM_BP_DIRECT	GO:0030218~erythrocyte differentiation	5	2.304147	9.37E-04
GOTERM_BP_DIRECT	GO:0030097~hemopoiesis	5	2.304147	0.003971
GOTERM_BP_DIRECT	GO:0043066~negative regulation of apoptotic process	13	5.990783	0.004454
GOTERM_BP_DIRECT	GO:0008285~negative regulation of cell proliferation	11	5.069124	0.012111
GOTERM_BP_DIRECT	GO:0000122~negative regulation of transcription from RNA polymerase II promoter	16	7.373272	0.012984
GOTERM_BP_DIRECT	GO:0007050~cell cycle arrest	6	2.764977	0.019582
GOTERM_BP_DIRECT	GO:0008584~male gonad development	5	2.304147	0.019829
GOTERM_BP_DIRECT	GO:0045944~positive regulation of transcription from RNA polymerase II promoter	19	8.75576	0.022256
GOTERM_BP_DIRECT	GO:0043161~proteasome-mediated ubiquitin-dependent protein catabolic process	7	3.225806	0.024531
GOTERM_BP_DIRECT	GO:0006810~transport	9	4.147465	0.03818
GOTERM_BP_DIRECT	GO:0030308~negative regulation of cell growth	5	2.304147	0.044148
GOTERM_BP_DIRECT	GO:0043065~positive regulation of apoptotic process	8	3.686636	0.047575
GOTERM_CC_DIRECT	GO:0005829~cytosol	63	29.03226	2.93E-06
GOTERM_CC_DIRECT	GO:0030863~cortical cytoskeleton	6	2.764977	3.03E-06
GOTERM_CC_DIRECT	GO:0005833~hemoglobin complex	4	1.843318	2.47E-04
GOTERM_CC_DIRECT	GO:0016020~membrane	39	17.97235	0.001861
GOTERM_CC_DIRECT	GO:0005737~cytoplasm	76	35.02304	0.001901
GOTERM_CC_DIRECT	GO:0080008~Cul4-RING E3 ubiquitin ligase complex	3	1.382488	0.013939
GOTERM_CC_DIRECT	GO:0005667~transcription factor complex	7	3.225806	0.017789
GOTERM_CC_DIRECT	GO:0016323~basolateral plasma membrane	6	2.764977	0.044445
GOTERM_CC_DIRECT	GO:0005856~cytoskeleton	9	4.147465	0.046641
GOTERM_MF_DIRECT	GO:0005515~protein binding	124	57.14286	9.16E-05
GOTERM_MF_DIRECT	GO:0051537~2 iron, 2 sulfur cluster binding	4	1.843318	0.002571
GOTERM_MF_DIRECT	GO:0008134~transcription factor binding	9	4.147465	0.014121
GOTERM_MF_DIRECT	GO:0042803~protein homodimerization activity	16	7.373272	0.015948
GOTERM_MF_DIRECT	GO:0003714~transcription corepressor activity	7	3.225806	0.025659
GOTERM_MF_DIRECT	GO:0004842~ubiquitin-protein transferase activity	9	4.147465	0.030192
KEGG_PATHWAY	hsa05144: Malaria	5	2.304147	0.003013
KEGG_PATHWAY	hsa04976: Bile secretion	5	2.304147	0.010206
KEGG_PATHWAY	hsa00860: Porphyrin and chlorophyll metabolism	4	1.843318	0.014778
KEGG_PATHWAY	hsa04141: Protein processing in endoplasmic reticulum	7	3.225806	0.017796
KEGG_PATHWAY	hsa00910: Cell Cycle	3	1.382488	0.018195
KEGG_PATHWAY	hsa05211: Renal cell carcinoma	4	1.843318	0.047755

### Identification of hub genes by PPI construction

After screening the interested green module, the PPI network was constructed by STRING database, and then it was uploaded into Cytoscape software to further construct sub-network and identify hub genes.

**Figure 8:**
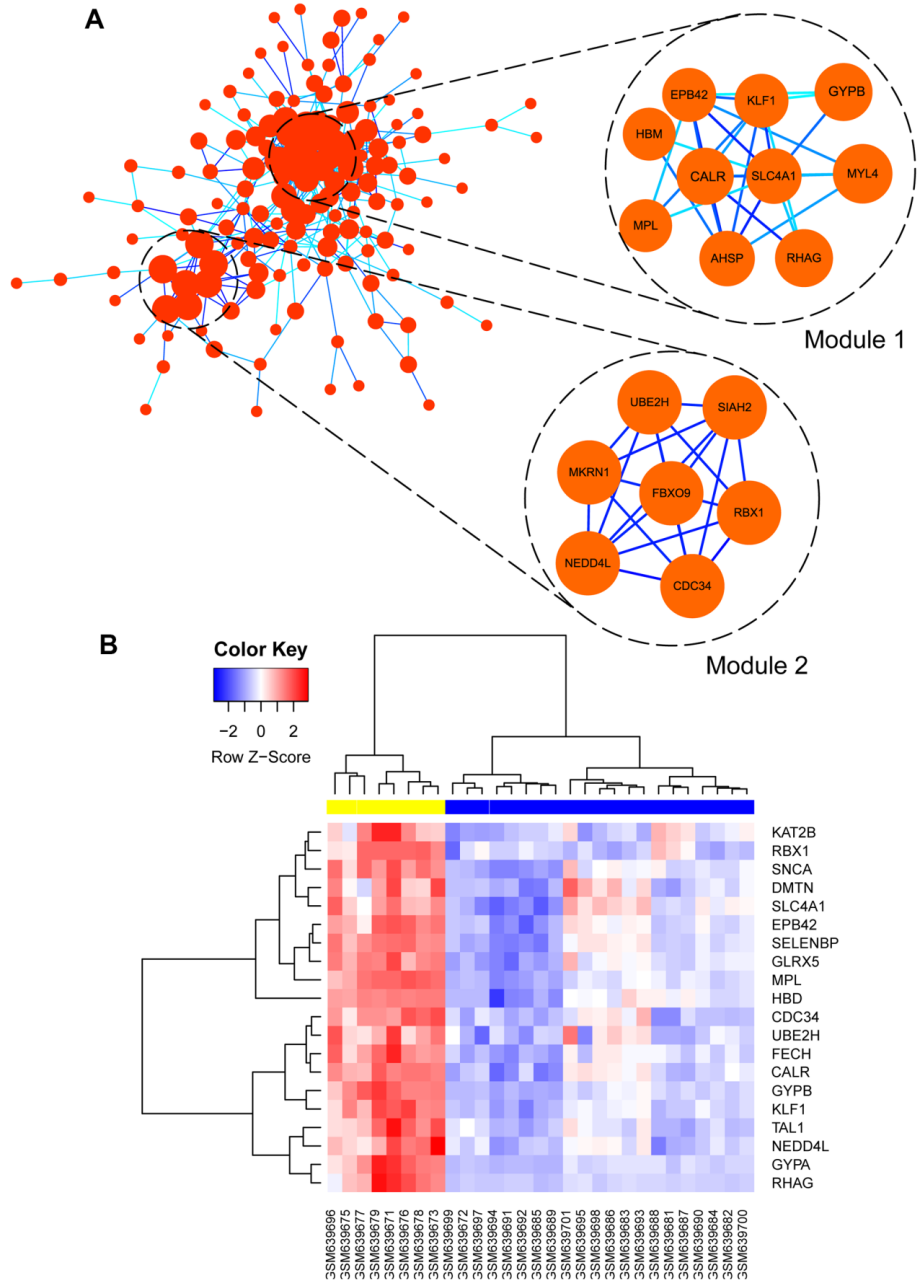
(**A**) Visualization of protein-protein interaction (PPI) network of genes in green module as well as the top 2 modules from PPI network. Every edge represented the interaction between two genes. (**B**) hub genes expression heat map identified in green module.

Co-related sub-modules in green module were also identified by MCODE plug-in in Cytoscape, totally 155 nodes and 863 edges were acquired, together with the top 2 significant sub-modules, as shown in Figure 8A. Module 1 contained 10 nodes and 55 edges, module 2 contained 7 nodes and 30 edges. Functional annotation and pathway enrichment of screened modules were conducted again using DAVID database. As Table 2 showed, results suggested that genes in module 1 were mainly focused on hemoglobin metabolic process, erythrocyte differentiation, iron ion binding and hemoglobin complex; genes in module 2 were primarily enriched in protein polyubiquitination, protein binding and cytoplasm, etc.

**Table 2:** Functional and pathway enrichment analysis of MCODE identified genes.

**Module**	**Term**	**Count**	**%**	**P Value**
1	GO:0020027~hemoglobin metabolic process	5	50	0.002856
	GO:0030218~erythrocyte differentiation	4	40	0.018902
	GO:0005833~hemoglobin complex	4	40	0.005912
	GO:0005887~integral component of plasma membrane	3	30	0.027505
	GO:0005506~iron ion binding	2	20	0.078693
2	GO:0000209~protein polyubiquitination	5	71.42857	2.06E-07
	GO:0016567~protein ubiquitination	4	57.14286	1.85E-04
	GO:0005737~cytoplasm	5	71.42857	0.025964
	GO:0005654~nucleoplasm	4	57.14286	0.027962
	GO:0004842~ubiquitin-protein transferase activity	7	100	5.24E-11
	GO:0005515~protein binding	7	100	0.019848

Subsequently, hub genes in this weighted network were filtered with degrees ≥ 36, degrees meant the level of correlation between two genes. Altogether, 20 genes were identified as hub genes including EPB42, SLC4A1, CALR, MPL, FECH, GYPB, KLF1, DMTN, RBX1, HBD, GYPA, GLRX5, UBE2H, KAT2B, RHAG, SELENBP1, CDC34, TAL1, NEDD4L, and SNCA, as listed in Table 3. Meanwhile, the gene expression levels of the hub genes between PMF and normal samples were tested, as shown in Figure 8B. Illustration elucidated that expression levels of these hub genes in PMF samples were much higher compared to normal samples, indicating that these genes were responsible for the development of PMF. Additionally, EPB42, CALR, SLC4A1 and MPL associated genes including HBM, KLF1, GYPB, MYL4, AHSP, RHAG were also expressed abnormally, which demonstrated that EPB42, CALR, SLC4A1 and MPL associated pathways were activated aberrantly.

**Table 3:** Detailed information of the hub genes screened in green module.

**Gene symbol**	**Degree**	**Betweenness**	**Gene symbol**	**Degree**	**Betweenness**
CALR	68	0.178851	GYPA	40	0.013157
EPB42	63	0.171656	GLRX5	38	0.048597
MPL	62	0.090448	UBE2H	38	0.104637
SLC4A1	53	0.026492	KAT2B	38	0.106015
FECH	52	0.073644	RHAG	37	0.00664
GYPB	52	0.01537	SELENBP1	37	0.050267
KLF1	46	0.013714	CDC34	36	0.025117
DMTN	43	0.077598	TAL1	36	0.080359
RBX1	43	0.050277	NEDD4L	36	0.048756
HBD	41	0.004011	SNCA	36	0.093156

Among those genes, the node degree of genes EPB42, CALR, SLC4A1 and MPL ranked highest, suggesting that EPB42, CALR, SLC4A1 and MPL had the most links with other proteins, which indicated that they were pivotal in the pathogenesis and oncogenesis of PMF. Consequently, these four genes were regarded as hub genes of PMF.

### Verification of expression patterns and protein expression of hub genes

**Figure 9:**
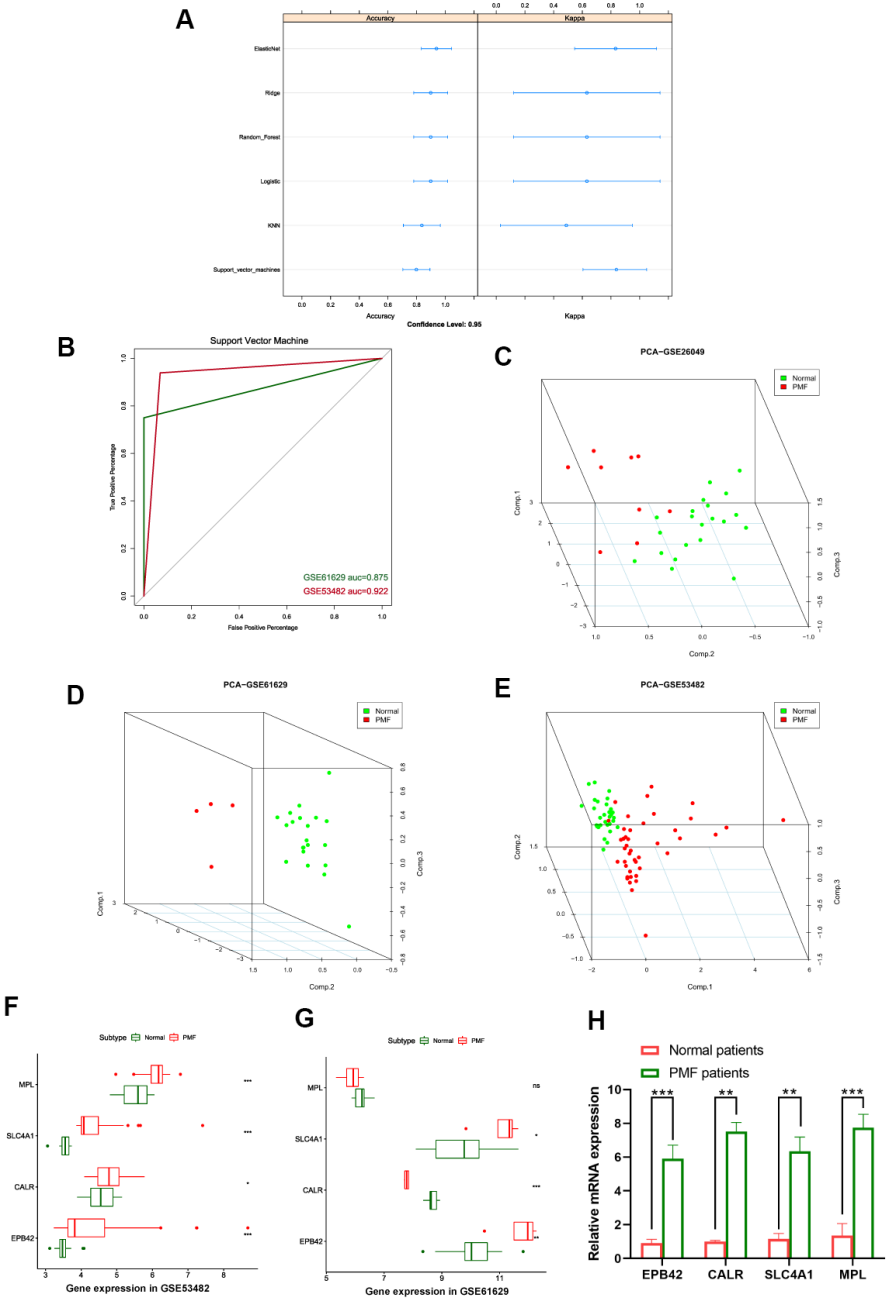
(**A**) Summary plot of different machine learning models. (**B**) ROC curves of support vector machine. (**C**–**E**) 3D scatter plot after principal component analysis of hub genes. (**F**) Expression values of hub genes between different groups in GSE53482. (**G**) Expression values of hub genes between different groups in GSE61629. ***: P < 0.001, **: P < 0.01, *: P < 0.05, ns: P> 0.05. (**H**) Expression of the hub genes EPB42, CALR, SLC4A1, MPL between PMF patients and healthy donors collected at our institution.

After EPB42, CALR, SLC4A1 and MPL were finally identified as hub genes of PMF, we established machine learning model to confirm the reliability of these genes, learning methods included Elastic Net regression, Ridge regression, Logistic regression, Random Forest, K-nearest neighbors, and Support vector machine models. Each of the machine model displayed high accuracy in Figure 9A. The most appropriate predicting model SVM (Support vector machine) was chosen as appropriate model and applied for testing sets in third party datasets, results were presented in Figure 9B, auc of GSE53482 and GSE61629 were 0.922 and 0.875, respectively.

Principal component analysis (PCA) was subsequently performed to reduce dimension of these hub genes so that we could observe the spatial distribution and clustering properties of the data. After dimension reduction, three principal components PC1, PC2 and PC3 were obtained, results displayed that these three components of the hub genes could differentiate the normal samples from PMF samples clearly in three-dimensional cube, from GSE26049, GSE61629, GSE53482, respectively (Figure 9C–9E).

Expression values of these four hub genes in different third-party GEO datasets were further analyzed, we verified the expression of EPB42, CALR, SLC4A1 and MPL among normal and PMF patients from GSE53482 and GSE61629 datasets (Figure 9F, 9G), results elucidated the hub genes were significantly overexpressed in PMF patients in both GSE53482 and GSE61629 series (P < 0.05). To further validate the expression of these hub genes, we collected the peripheral blood from healthy donors and PMF patients from our institution, and conducted qRT-PCR analysis. The results, presented in Figure 9H, suggested a significant difference between normal and PMF patients among these hub genes (P < 0.05).

**Figure 10:**
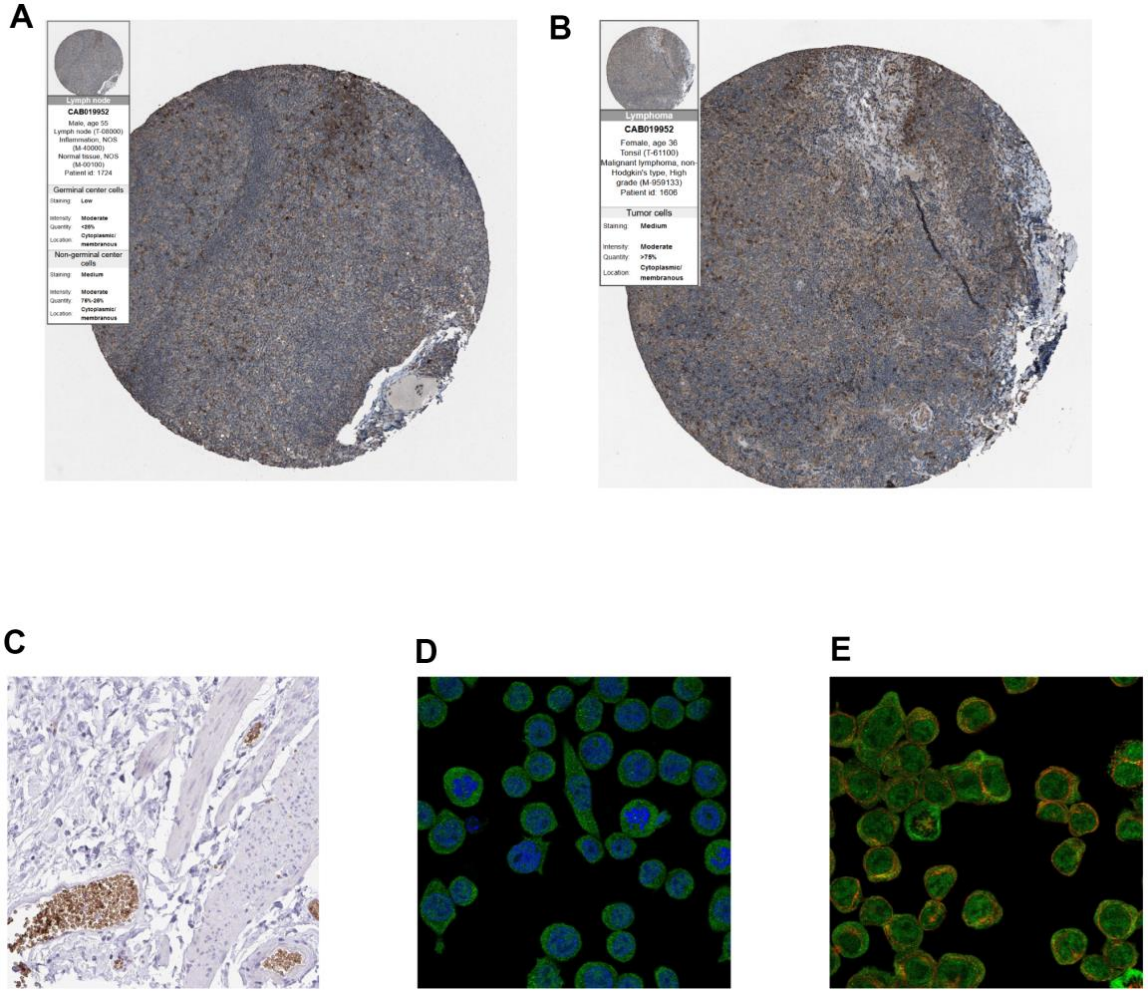
(**A**, **B**) Immunohistochemistry of CALR gene in normal and tumor tissues from HPA database. (**A**) Normal tissues (antibody: CAB019952; staining: low; intensity: moderate; quantity: < 25%). (**B**) Tumor samples (antibody: CAB019952; staining: medium; intensity: moderate; quantity: > 75%). (**C**) Immunohistochemical staining of human gallbladder shows positivity in erythrocytes, (original magnification: 20x). (**D**, **E**), Immunofluorescence of EPB42 gene in HEL cell lines (myeloid tissues, antibody: HPA040261) (original magnification: 400x).

**Figure 11:**
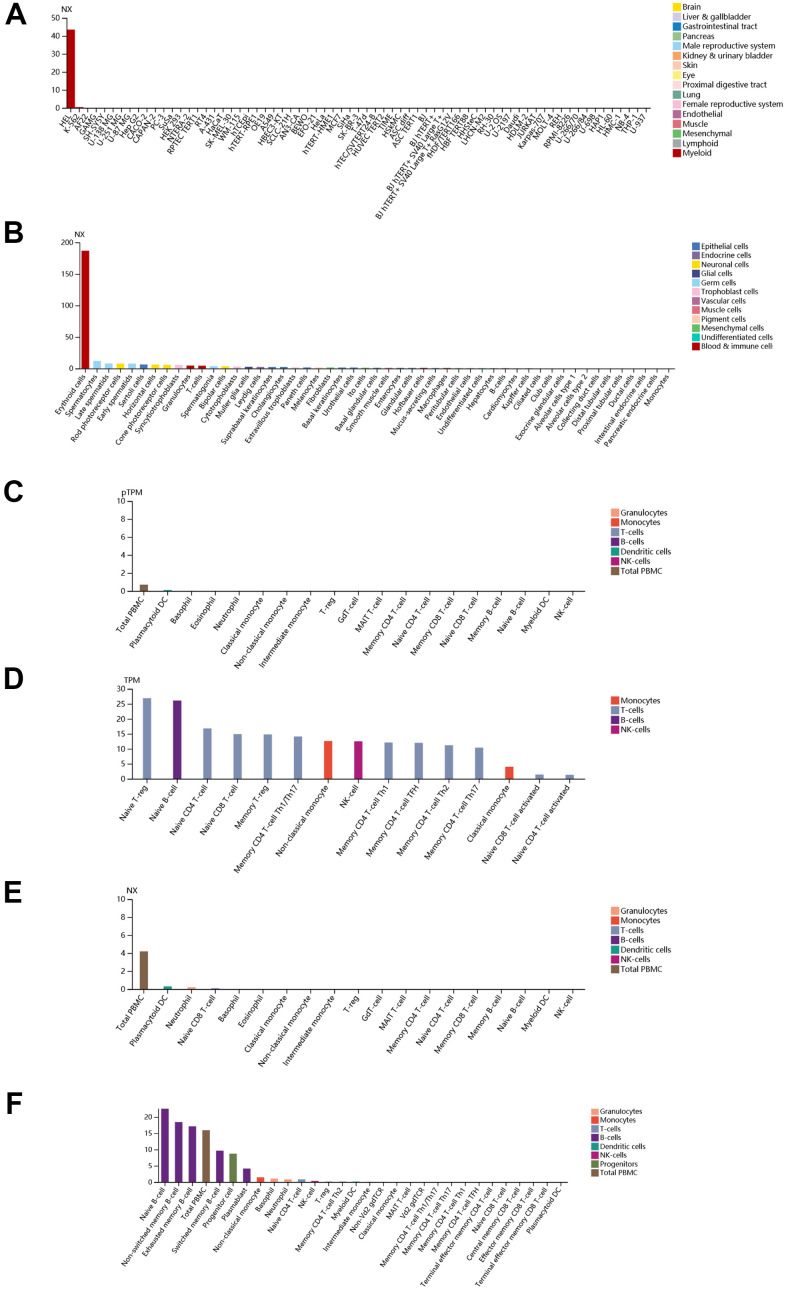
**Detailed expression information of EPB42 in different kinds of cells and tissues.** (**A**) Expression situation of EPB42 in different tissues; (**B**) Expression situation of EPB42 in different kinds of cells; (**C**–**F**) Expression situation of EPB42 in different immune cells.

Finally, immunohistochemistry (IHC) as well as immunofluorescence (IF) were performed to detect relative location and abundance of proteins. The protein level of CALR gene was significantly overexpressed in tumor tissues compared to normal tissues based on HPA database (Figure 10A, 10B). The protein expression location of EPB42 was highly expressed in myeloid tissues as well as erythrocytes in gallbladder, results suggested that they were enriched in nucleoplasm and cytosol (Figure 10C–10E). The detailed expression information of EPB42 in different kinds of cells and tissues could be known in Figure 11, they were highly expressed in HEL cell lines, and Peripheral blood mononuclear cell (PBMC).

## DISCUSSION

PMF, one of the most complex and rare malignant tumors in MPNs, is frequently but not always accompanied by JAK2, CALR mutation, aberrant cytokine expression, bone marrow fibrosis and anemia, etc. [25–27]. Even though great progress has been made in the diagnosis of MPN during the last decades, the relevant research of PMF, is not sufficient, overall treatment and prognosis of PMF is still unsatisfactory, followed by a short median survival time as well as an inadequate exploration of chemotherapy [10–13]. This situation may be blamed for lack of effective biomarkers for targeted therapy. Consequently, to better discover novel biomarkers that could effectively and accurately predict the progression of PMF, this study extracted gene expression profile of GSE26049 from GEO database, and made a holistic WGCNA analysis.

WGCNA, a powerful global research tool for data mining from multiple genes in large-scale datasets, is developed rapidly in recent years, which is characterized by filtering significant modules from phenotype features and clinical traits. WGCNA has already been used in recent years and has made a great progress in the identification of biomarkers and targeted chemotherapy [28–32]. Therefore, WGCNA is a reliable analysis tool, to our knowledge, whether the application of WGCNA methods or related research in PMF has rarely been reported. As a result, a comprehensive understanding in the pathogenesis of PMF at the early stage is imperative, to provide effective therapeutic strategies.

In the present study, 14 co-expression modules were generated based on 20482 genes from 89 samples in GEO database by WGCNA. This study aimed to elucidate the association between modules and PMF feature and excavate the real biological meaning behind hub genes. First, hierarchical clustering analysis was generated to detect outliers and cutoff threshold was set as 85 to eliminate the outliers. Next, the optimal soft threshold power was picked by “pickSoftThreshold” function in R to construct a scale-free topology network, which could get access to the real biological network state. Soft threshold power was a key value to amplify the disparity among strong and weak correlation genes, which could affect the mean connectivity and the scale independence of co-expression modules. Ultimately, soft threshold power β was determined as 16.

After the weighted co-expression network was constructed based on soft threshold power, hierarchical clustering was performed to visualize the subgroups of weighted network. Altogether, 14 distinct co-expression modules were generated, and each module contained the genes that had the most correlations with each other. After acquiring WGCNA network data, the module-trait heatmap was displayed, we observed that green module had the strongest positive correlation to PMF group, illustrating those genes in green module were crucial in the pathogenesis of PMF. Meanwhile, purple and yellow modules had a highly positive correlation in normal group, indicating that genes in these modules may benefit for the normal organism development. Additionally, the correlation in PV and ET groups were not as significant as PMF group (Figure 4A). Besides, hierarchical clustering analysis in green module visualized that genes expression level could significantly differentiate PMF group from other three groups (Figure 5C). These findings implied, consistent with previous studies, that PMF is the most major neoplasms in MPNs, later in their courses, both PV and ET disorders may be evolved into post-PV MF and post-ET MF due to gene mutation or over-expression [3, 4].

GO, KEGG and GSEA methods were then carried out to further study the function and pathway regulation mechanisms of PMF tumorigenesis. Functional annotation revealed that genes in green module were mainly enriched in erythrocyte differentiation, hemoglobin complex, transcription factor complex and protein binding, which may explain the reason why the fast multiplication of cancer cells and the generation of tumor cells. Our results implied that abnormal changes such as erythrocyte differentiation may occur in hemoglobin complex, which agreed with previous studies that myeloproliferative differentiation disorders may lead into aplastic anemia in the progression of PMF [33]. Results also found that molecular functions were primarily enriched in transcription factor binding in transcription factor complex, which was consistent with recent studies that cytokines mediated corresponding receptors into the nucleus to participate in transcriptional regulation, and finally lead into tumor growth [5]. Furthermore, the analysis of KEGG and GSEA revealed that these genes were primarily enriched in cell cycle, hematopoietic cell lineage, JAK-STAT signaling pathway, oocyte meiosis, and P53 signaling pathway. The oocyte meiosis was firstly contacted to PMF, and the mechanism was presumed to be related to progesterone-mediated oocyte maturation, which was reported to be related with many neoplasms [34]. Next, toll-like receptor signal pathway was found to be highly activated in normal samples, indicating that toll-like receptor behaved in resisting tumor in normal individuals. Besides, protein processing in endoplasmic reticulum, bile secretion and porphyrin metabolism were also aberrantly activated in PMF, the reason may be due to hepatomegaly and hyperfunction caused by genes over expression. Advanced researches reported that porphyria was a group of porphyrin metabolic disorders caused by the deficiency or the decrease of special enzyme in the pathway of heme synthesis. Based on the findings in this study that module genes of PMF may result in in heme binding, porphyrin metabolism, etc., we have reason to deduce that the development of PMF may also lead to the occurrence of porphyria, further research needs to be conducted to find out the correlations between these two disorders. Additionally, existed studies also reported that dysregulation of cell cycle may result in the oncogenesis of tumor [35, 36], which was agreed with our findings that cell cycle was aberrantly activated in PMF.

With the aim of screening hub genes among green module, the interaction network of 217 mutual genes were constructed by PPI based on the STRING database. Altogether, 20 genes were selected with high degrees. Heatmap of these genes’ expression between PMF samples and normal samples illustrated that these genes could significantly distinguish these two groups (P < 0.05, Figure 8B). Particularly, EPB42, CALR, SLC4A1 and MPL, namely hub genes of PMF, ranked highest in their degree value, indicating that these genes had the most correlations with the progression of PMF.

To further test the reliability of these hub genes, we established a series of methods including machine learning models, PCA analysis, qRT-PCR, and HPA database validation, etc. Machine learning is a method of summarizing rules, extracting information, and finally predicting unknown results, ROC curves generated by SVM suggested a good predicting ability, demonstrating that these hub genes had a pretty classification ability between normal and tumor samples. PCA analysis visualized that based on the three principal components, these hub genes could make a good distinction between different samples in three-dimensional cube. Furthermore, the hub genes were all highly expressed in PMF samples than normal samples based on GEO database. HPA database including IHC and IF also proved the aberrant expression situation of the four hub genes. To confirm our findings, the results of our *in-silico* analysis were compared with qRT-RCR data from cases collected at our institution. Consistent with the *in-silico* data mining results, these four hub genes EPB42, CALR, SLC4A1 and MPL were all overexpressed in PMF patients than that in healthy donors (P < 0.05). Consequently, these results mentioned above proved that the hub genes had a high correlation with PMF. While these differences were significant, the sample size were not large enough at this moment, due to the rare and difficult to collect of PMF, thus future validation about more samples at our institution would be required.

CALR, located on chromosome 19, namely calreticulin, was a multifunctional protein that acted as a major Ca^2+^ binding protein in the lumen of the endoplasmic reticulum. Advanced researches proved that it also existed in nucleus, suggesting that it may activate in transcription regulation. Many studies had reported their highly correlation with the development of MPNs [5, 37, 38], driver mutations involving CALR in 90% of patients mediated JAK-STAT signaling pathway, thereby leading to changes in the cytokine and growth factor milieu and accordingly potentiated fibrosis and finally resulted in disease. The immunohistochemistry results were consistent with the previous findings that CALR were promoted in the progression of MPNs [39], this study further proved that CALR was mainly associated with PMF. MPL, located on chromosome 1, mediated the expression of thrombopoietin and its receptor, activated in cellular signaling pathway, which were pivotal to the expansion and regulation of megakaryocytes as well as self-renewal of hematopoietic stem cells. Previous researches showed that mutations in both MPL and CALR could increase the JAK-STAT activation, of which CALR may induce JAK-STAT signaling pathway by increasing recruitment of proteins to the MPL promoter site [40].

EPB42, namely erythrocyte membrane protein band 4.2, was an ATP-binding protein which may activate the correlation of protein 3 with ankyrin. It was reported that EPB42 could control the shape and mechanical property of erythrocyte, and it had a high association with hereditary spherocytosis and recessive inherited hemolytic anemia [41, 42]. Currently, EPB42 was rarely reported in literature, this study proved the correlation between EPB42 and PMF. SLC4A1, part of the anion exchanger family and was expressed in the erythrocyte plasma membrane. The expressed protein comprised two domains which were structurally and functionally distinct, of which the N-terminal 40kDa domain was in the cytoplasm and acted as an attachment site for erythrocyte skeleton by binding with ankyrin. Many mutations in this gene had been known in human, and it was reported to be that over expression of SLC4A1 could lead to some diseases like hereditary spherocytosis caused by destabilization of cell membrane, and inherited distal renal tubular acidosis [43, 44]. The characteristics that EPB42 and SLC4A1 was over expressed in hereditary spherocytosis was consistent with the hematological features of PMF that the presence of large amount of erythrocyte in the peripheral blood cell smear. Besides, positive staining of EPB42 in erythrocyte and the expression of EPB42 in PBMC also demonstrated a high correlation with blood diseases. Based on the findings above, this study found EPB42 and SLC4A1 had a strong correlation with PMF, immunofluorescence of EPB42 proved our results in this study, which were detected highly expressed in tumor samples. Consequently, our results discussed the connections between the hub genes EPB42, CALR, SLC4A1, MPL and PMF together with other related blood diseases comprehensively.

Overall, this study used WGCNA analysis for a whole situation to conduct an elaborate, precise, and systematic view to analyze the DEGs from PMF disease. Currently, there is still lack of specific diagnostic indicators for malignant tumor in hematological diseases. EPB42 and SLC4A1, served as a biological macromolecule, which received little attentions in studies, have been proved in this study to be new biomarkers of PMF, the effects are worth expected whether in the diagnosis at early stage or as therapeutic target. However, considering all the results above, there were still some limitations, molecular biological experiments and more large patient samples need to be applied in the future to further validate these hub genes, and to determine whether they may be beneficial in the diagnosis or treatment of PMF.

## CONCLUSIONS

This study attempted to explore the potential molecular regulatory mechanism of PMF based on WGCNA analysis. 14 distinct co-expression modules were identified from GEO dataset GSE26049. Of which, green module was most significantly correlated with PMF. EPB42, CALR, SLC4A1 and MPL in green module were recognized as key therapeutic targets for PMF. EPB42 and SLC4A1, which were rarely reported in literature, have been proved to be highly correlated with PMF.

## MATERIALS AND METHODS

### Microarray data

Gene expression profile of GSE26049 was downloaded from the National Center for Biotechnology Information Gene Expression Omnibus (GEO, https://www.ncbi.nlm.nih.gov/geo/), which is a functional public genomics repository, including high throughput gene expression data, chips, and microarray data. The profile totally contained 90 samples, including 9 PMF (primary myelofibrosis) samples, 19 ET (essential thrombocythemia) samples, 41 PV (polycythemia vera) samples and 21 normal samples.

### Gene expression profiles’ preprocessing

The method of quality control was performed by R (“affy”, “affyPLM” package), standard samples were identified through calculating their normalized unscaled standard error (NUSE). Data preprocessing was performed to handle the raw data (“.CEL” file format) using robust multi-array average (RMA) background correction and normalization (RMA function, “affy” and “affyPLM” package). The Affymetrix annotation files from GPL570 platform were applied to annotate corresponding probes, and probes without annotation were removed.

### Weighted gene co-expression network analysis (WGCNA)

WGCNA analysis was performed to identify corresponding expression modules. The process were conducted under R environment (“WGCNA” package [18]). First, hierarchical clustering analysis was applied to check the heterogeneity of samples to detect and eliminate the outliers. Second, clustering was performed according to gene expression levels in each sample to construct the network, to get access to the real biological network state (scale-free network). The soft threshold power was determined to select by computing a correlation value between every pair of genes, which could amplify disparity among strong and weak correlations, so that the network state could approach scale-free network topology. The optimal soft threshold power was selected when the scale-free topology index (R^2) reached 0.90 and mean connectivity approached 0.

Next, weighted gene co-expression network was constructed based on the correlations of gene expression levels, and co-expression modules were identified by dynamic pruning method. The minimum number of genes in module was set as 30. Eigengenes adjacency was calculated to evaluate interaction of various gene modules. Module-trait relationships were estimated to identify the correlation between modules and PMF.

The degree of correlation between genes was calculated by WGCNA using the Topological overlap measure (TOM) [45, 46], and Pearson’s correlation coefficient was also calculated to evaluate correlation between module eigengene and phenotype. P < 0.01 was regarded as statistically significant. Then relationships between Gene Significance (GS) and Module Membership (MM) was assessed. Ultimately, the module with the highest weighted correlation coefficient among all clustered modules was chosen as the interested module, and was pooled for further analysis.

### Functional and pathway enrichment analysis on interested module

Functional annotations and interpretations were applied to discover their biological meaning behind genes. This study used DAVID database (Database for Annotation, Visualization, and Integrated Discovery, http://david.ncifcrf.gov/), to get comprehensive understanding, which is a crucial repository providing a set of functional annotation tools, to explore the real biological significance. GO (Gene Ontology) is a powerful tool to analyze the molecular functions (MFs), cellular components (CCs), and biological processes (BPs) of genes. KEGG (Kyoto Encyclopedia Genes and Genomes) pathway enrichment analysis was performed to understand links among different genes and signaling pathways. This study performed GO and KEGG in DAVID database and P < 0.05 was set as the cutoff threshold. Additionally, GSEA (Gene set enrichment analysis) was further conducted to determine which set of genes showed statistical significance. Based on the size of set, a normal enrichment score (NES) was assessed for each gene set, FDR and P value were calculated as the cutoff criteria.

### PPI (protein-protein interaction) network construction and hub genes selection

PPI analysis was then conducted by STRING online tool, (Search tool for retrieval of interacting genes, https://string-db.org/), an online database that provided PPI correlations. Subsequently, we used Cytoscape software (version 3.7.0) to screen hub genes and modules through Molecular Complex Detection (MCODE) plug-in, which was used for clustering given network links based on topology to identify densely connected regions. Each node in the network represented a gene, and edges represented regulatory relationships between genes. MCODE recognized the most significant modules through mathematical algorithm that number of nodes > 7 and MCODE scores > 8. P < 0.05 was regarded as statistical significance and hub driver genes were selected as degrees ≥ 36.

### Validation of hub genes in different GEO databases

The identified hub genes were validated in third party GEO database: GSE53482 and GSE61629, which were set as testing sets. The gene expression profiles were downloaded from GEO database, and annotation file were provided by platforms “GPL570, Affymetrix Human Genome U133 Plus 2.0 Array” for GSE61629 and “GPL13667, Affymetrix Human Genome U219 Array” for GSE53482. The initial downloaded file was “.CEL” format, “RMA” algorithm by R (“rma” function) was conducted to perform background correction and data normalization to obtain standard gene expression profiles, followed by probe annotation. Based on manufacture-provided annotation files on platform, probe sets without corresponding genes were removed. The expression of hub genes in these GEO datasets were analyzed between normal group and PMF group to validate the significance of these genes.

### Establishment of machine learning and principal component analysis

To test the predictive ability and reliability of these hub genes, GSE26049 was set as training set, then different machine learning algorithms were constructed based on training set, including Elastic Net regression, Ridge regression, Logistic regression, Random forest, K-nearest neighbors, and Support vector machine algorithms (“caret”, “e1071”, “kernlab” packages were needed). Five-fold cross validation was set to obtain the most suitable equation as well as the most accurate predicting results. GSE53482 and GSE61629 were set as testing sets to confirm the accuracy of these machine learning models, ROC curve of the most suitable model was plotted and auc (area under curve) was calculated. Subsequently, based on the hub genes expression, principal component analysis (PCA) was conducted to reduce dimension to observe whether these hub genes could distinguish the tumor samples from the normal samples.

### Validation of protein expression of the hub genes by HPA database

The hub genes between PMF and normal samples in this study were determined using immunohistochemistry (IHC) as well as immunofluorescence (IF) by Human protein atlas database (https://www.proteinatlas.org/). HPA is a valuable repository which provides a large-scale of protein and transcriptomic data in specific human tissues and cells for researchers to visualize the most valuable information [47]. In addition, the IHC-based protein expression pattern is the best way to detect the protein location and abundance of interested genes.

### Real-time qRT-PCR in PMF and normal patients

To confirm the expression situation of these four hub genes in PMF patients, this study collected peripheral blood tissues from totally 4 PMF patients and 4 healthy donors between December 1, 2020 and September 10, 2021 in Xi’an Daxing hospital. Total RNA was extracted from whole-blood using the Paxgene Blood RNA Kit (qiagen762174), and qRT-PCR was performed using FastStart Universal SYBR Green Master (ROX) (Roche Diagnostics) in a CFX96 Real-Time System (Bio-Red) according to the manufacturer’s instructions, and expression levels were normalized to glyceraldehyde-3-phosphate dehydrogenase (GAPDH). The 2^-ΔΔCt^ method was applied for qRT-PCR data analysis. The primers of corresponding genes were listed as follows: EPB42 forward primer, ACATGTCAGGGTGCTCACAG; EPB42 reverse primer, TTGCTTCTGGGCTCCTTCTG; CALR forward primer, CGCTTTTATGCTCTGTCGGC; CALR reverse primer, CCACAGATGTCGGGACCAAA; SLC4A1 forward primer, CACACAACTTCAGGCCCCTC; SLC4A1 reverse primer, AGAGCCTGCTGTCTCCTACC; MPL forward primer, TCTCCTCTTCTAGCATTTCTTCCA; MPL reverse primer, AGCATCACAGTGCTGTAGTAGA; GAPDH forward primer, GACAGTCAGCCGCATCTTCT; GAPDH reverse primer, GCGCCCAATACGACCAAATC. This research was approved by the Ethics Committee of Xi’an Daxing hospital. All participating patients and donors have understood the experimental procedures and signed the informed consents.

### Data availability statement

The data used and analyzed during the current study are available upon reasonable request.

### Consent for publication

All contributing authors agree to the publication of this article.

## Supplementary Material

Supplementary Table 1
